# The Mediating Effect of Negative Emotion on the Well-Being Consequence of Belongingness Deficit Across Ages

**DOI:** 10.1093/geroni/igaa057.1487

**Published:** 2020-12-16

**Authors:** Vivian Hiu Ling Tsang, Helene Fung

**Affiliations:** 1 The Chinese University of Hong Kong, Hong Kong, China; 2 The Chinese University of Hong Kong, Sha Tin, China

## Abstract

Previous studies suggested that the negative influence of belongingness deficit on wellbeing may be driven by an increase in negative emotion, but the age difference of this mediating effect is still uncertain. This study tested the mediating effect of negative emotion in the influence of relationship quality on depressive symptoms across age. Based on the potential biological decline in emotional sensitivity and better management of emotion with age, we hypothesize that negative emotion may be a weaker mediator in driving the consequences of relationship quality among older adults than younger adults. A total of 494 participants (19 – 85 years old, 60% was female) participated in this study at three timepoints with a two-year interval across Hong Kong, USA and Germany. Results suggested a significant partial mediating effect of negative emotion (b = -.0920, 95%LLCI = -.1315 to 95%ULCI = -.0568). In particular, poorer relationship quality at timepoint 1 predicted more negative emotion at timepoint 2 and therefore predicted more depressive symptoms at timepoint 3. More importantly, the mediating effect was moderated by age significantly (b = .0025, 95%LLCI = .0009 to 95%ULCI = .0042). As expected, the mediating effect of negative emotion decreased with age and became not significant among individuals at older age (b = -.0344, BootLLCI = -.0805 to BootULCI = .0075). Future studies can further investigate on age-related mediators in driving the consequences of belonginess deficit.

